# Mediterranean Diet and its Effect on Endothelial Function: A Meta-analysis and Systematic Review

**DOI:** 10.1007/s11845-022-02944-9

**Published:** 2022-02-22

**Authors:** Kaneez Fatima, Ahmed Mustafa Rashid, Usama Abdul Ahad Memon, Syeda Sidra Fatima, Syed Sarmad Javaid, Omema Shahid, Fazila Zehri, Muhammad Adil Obaid, Mahlika Ahmad, Talal Almas, Abdul Mannan Khan Minhas

**Affiliations:** 1 Department of Medicine, Dow University of Health Sciences, Karachi, Pakistan; 2 Department of Medicine, Jinnah Sindh Medical University, Karachi, Pakistan; 3 Department of Medicine, Ziauddin Medical University, Karachi, Pakistan; 4 Royal College of Surgeons in Ireland, Dublin, Ireland; 5 Department of Internal Medicine, Forrest General Hospital, Hattiesburg, MS USA

**Keywords:** Cardiovascular disease, Endothelial function, Flow-mediated dilation, Mediterranean diet

## Abstract

**Background:**

Endothelial dysfunction serves as an early marker for the risk of cardiovascular disease (CVD); therefore, it is a site of therapeutic interventions to reduce the risk of CVD.

**Aims:**

To examine the effect of the Mediterranean diet (MedDiet), as an intervention, on structural and functional parameters of endothelial function, and how it may reduce the risk of CVD and associated mortality.

**Methods:**

Medline database was searched for randomized controlled trials. Random-effects meta-analysis was conducted on 21 independent datasets. Meta-regression and subgroup analysis were performed to assess whether the effect of MedDiet was modified by health status (healthy subjects or with increased CVD risk), type of MedDiet intervention (alone or combined), type of parameter (functional or structural), study design (cross-over or parallel), BMI, age, and study duration. Our study used sample size, mean, and standard deviation of endothelial function measurements for both MedDiet intervention and control in the analyses.

**Results:**

Inverse relationship between endothelial function and intake of MedDiet was observed (SMD: 0.34; 95% CI: 0.16, 0.52; *P* = 0.0001). Overall, MedDiet increased FMD by 1.39% (95% CI: 0.47, 2.19; *P* < 0.001). There was a significant improvement in endothelial function in both healthy patients and in those with an increased risk of CVD. No significant variation was observed in the effects of MedDiet on endothelial function, due to study design or type of intervention.

**Conclusions:**

These findings support that MedDiet can reduce the risk of CVD by improving endothelial function.

**Supplementary information:**

The online version contains supplementary material available at 10.1007/s11845-022-02944-9.

## Introduction

The endothelium is an intricate organ that maintains vascular homeostasis through interacting with endothelial cells and the lumen of blood vessels [1]. For example, thrombosis and the regulation of blood pressure (BP) are two domains that the endothelium maintains. Thrombosis is maintained by secreting procoagulants and anticoagulants, and blood pressure is regulated by sustaining a balance between vasodilators and vasoconstrictors to control vascular tone, all of which have an impact on the progression of cardiovascular disease (CVD) [2]. In contrast, endothelial dysfunction is defined as a change in the physiology of the endothelium due to an imbalance in the availability of active substances of endothelial origin. This makes the endothelium vulnerable to inflammation, vasoconstriction, and raised vascular permeability, leading to events such as platelet aggregation, thrombosis, and arteriosclerosis [1]. Loss of endothelial integrity, both functional and structural, plays a pivotal role in the development of atherosclerosis, progression of plaques, and subsequent atherosclerotic complications [3, 4]. Evidence suggests that endothelial dysfunction can serve as an early marker for those that are at a higher risk for vascular disease, as observed in a group of hypertensive patients [2]. Therefore, the endothelium serves as a physiological site for curative interventions for the reduction of risk factors of CVD such as stroke and coronary heart disease [5, 6]. Cardiovascular disease is among the leading causes of death globally, among which ischemic heart disease accounts for 16% of total world deaths [7].

One such intervention, the Mediterranean diet (MedDiet) represents dietary patterns of the Mediterranean region, which has a low incidence of cardiovascular death as compared to the other populations [8]. The pattern of the MedDiet has different levels of food consumption such as focusing on a high intake of olive oil, fruits, and nuts, whereas the intake of fish should be at a moderate-to-high level with a focus on low consumption of sweets [9, 10]. Olive oil is a popular ingredient of the MedDiet as it is known for its benefits, which are mainly credited to the mono-unsaturated fatty acids (MUFA), antioxidants, and anti-inflammatory compounds found within, that may be responsible for preventing chronic low-grade inflammation. This type of inflammation of the endothelium has been linked to events of CVD [11]. Additionally, data suggests that the risk of CVD is inversely associated with the intake of olive oil [12, 13]. While olive oil is an essential part of the MedDiet, it is the combination of foods within this diet that has been linked to better health [8]. The Lyon Diet Heart Study in France [14] and the Prevención con Dieta Mediterránea (PREDIMED) trial in Spain [15] showcased that MedDiet is effective in preventing primary and secondary CVD.

As evidenced from various randomized clinical trials (RCTs), it is proposed that different components of the MedDiet (olive oil [16], nuts [17], oily fish [18, 19]), even when consumed separately, can enhance endothelial function. Moreover, a compound MedDiet is observed to improve endothelial integrity in healthy individuals and those with cardiovascular and metabolic diseases. In a MedDiet intervention-based study, significant improvement in endothelial function in older healthy patients was observed, assessed using flow-mediated dilation (FMD) with an absolute rise of ~ 1.3% [20].

However, the number of clinical trials done to assess the effect of MedDiet on CVD and related outcomes remains small. There was one major trial, namely the PREDIMED [15], which showed improved outcomes but it was riddled with controversy. In an analysis of the trial published in 2019, multiple concerns were pointed out [21]. The intervention was not a composite MedDiet, but single food items. The control group did not necessarily consume a non-MedDiet. The primary outcome was a composite of three end-points. Effect sizes were probably overestimated due to an early stop to the trial after initial analyses indicated improved outcomes. In-depth observation also hinted towards lack of proper randomization [22]. All this contributed towards the trial being retracted and republished.

Due to this, the question remains whether MedDiet is good for cardiovascular outcomes. Therefore, to assess whether there is a physiological basis to support the claim, this study aims to conduct a meta-analysis on published RCTs to evaluate the effect of the MedDiet on structural (e.g., intima-media thickness) and functional (e.g., FMD) measures of endothelial function and how it subsequently reduces the risk of CVD and associated mortality.

## Methods

This meta-analysis was conducted according to the Preferred Reporting Items for Systematic Review and Meta-Analyses and the American Heart Association guidelines for systematic reviews [23].

### Literature search

Two reviewers (AMR, UAAM) independently searched MEDLINE from inception till 14th July 2021. No time or language restrictions were set. A detailed search strategy is provided in Supplementary Table S1. Our search also included databases of grey/unpublished literature as well as bibliographies of identified articles, clinical trial registries of ongoing or planned trials, recently published editorials from major medical journals, and reviews on the topic.

### Study selection

All the selected studies were imported to EndNote X9 (Thomson Reuters, Toronto, Ontario, Canada) and duplicates were identified and removed. The remaining studies were examined on title and abstract by the two reviewers, AMR and UAAM. The full text was appraised critically against the inclusion and exclusion criteria for the final selection of the articles. A third reviewer (SSJ) was consulted to resolve any discrepancies. No language restrictions were applied. Studies were included based on the following eligibility criteria: (1) randomized controlled trials; (2) studies with adults aged ≥ 18 years; (3) MedDiet (which was defined as a MedDiet by the authors of each study) administered alone or with any other intervention if a comparable and valid control group was present; and (4) studies reporting modifications in endothelial function for intervention and control groups separately. We excluded studies if they were case series, observational studies, or systematic reviews.

### Data extraction and quality assessment

Data were extracted by the first investigator (AMR) and then rechecked for accuracy by the second investigator (UAAM). Data extracted included study and population characteristics, and outcomes, including baseline and post-intervention values for endothelial function. In addition, two reviewers (AMR, UAAM) assessed the quality of the RCTs as low, high, or unclear risk of bias according to the Cochrane risk of bias tool for randomized controlled trials [24].

### Statistical analysis

Data were analyzed using RevMan software (Review Manager Version 5.3.5, The Nordic Cochrane Centre, Copenhagen). For our study, sample size, and the mean and standard deviation (SD) of the endothelial function measurements for pre and post-intervention periods (for both MedDiet intervention and control) were extracted and used in the analyses. When studies used more than 1 method to evaluate changes in endothelial function (Table 1), the compounding factor was taken into consideration by estimating the mean of the standardized effect sizes in order to prevent the overestimation of effect sizes. Effect sizes and 95% CIs for the MedDiet interventions were calculated using a weighted DerSimonian-Laird random-effects model [25]. Forest plots were generated to evaluate the compound effect of the Mediterranean diet on endothelial function. Two strata were made for endothelial function measurement, structural and functional. Functional measurements include FMD derived from ultrasound, forearm blood flow (FBF) derived from plethysmography, or cutaneous microcirculation derived from laser Doppler. Structural measurements included intima-media thickness or vessel size, both measured by ultrasound. FMD was used in the majority of the studies as it is a non-invasive, clinical measurement of endothelial function [26]; hence, sensitivity analysis was performed on the effects of MedDiet on FMD as a majority of the studies reported this value. Leave-one-out sensitivity analysis was conducted to assess if any single study disproportionately influenced the results and resulted in an increase in heterogeneity. Random-effects meta-regression analyses were carried out to check whether participants’ baseline characteristics (such as mean age and body-mass index (BMI)) and duration of studies included modified the impact of MedDiet on endothelial function. Meta-regression was also applied to check for association, if any, between study duration (in weeks) and functional and structural parameters of endothelial function, separately. We investigated the risk of publication bias by funnel plots and Egger’s regression test and statistically assessed by Begg's Test (*P* > 0.05 as no publication bias). Heterogeneity was assessed using the Cochrane *Q* statistic; *P* < 0.1 indicates significant heterogeneity. Heterogeneity across the trials was also evaluated by the *I*^2^ test and the scale was set as a value < 25% which indicates low risk; 25–75% indicates moderate risk; and > 75% indicates high risk [27]. A *p*-value of < 0.05 was considered significant in all cases. All data used in meta-analysis can be found in Supplemental Tables 1, 2, 3, 4 and 5.

**Table 1:** Characteristics of the randomized controlled trails included in the study

Author	Country	Study design	Health status	Outcome	Sample size	Age(y)	BMI (kg/m3)	SBP/DBP (mmHg)	Duration (week)	Intervention	Control
Ambring et al. [28]	Sweden	Crossover	Healthy	FBF	22	43	26	-	12	MedDiet	Swedish diet
Buscemi et al. [29]	Italy	Parallel	Obese	FMD	20	38	34.2	128/88	8	MedDiet	Atkins low carbohydrate diet
Ceriello et al. [30]	Spain	Parallel	DM2	FMD	24	-	29.5	116/78	12	MedDiet + MUFA	Low-fat diet
Davis et al. [20]	Australia	Parallel	Healthy	FMD	166	71	26.9	124/71	24	MedDiet	Habitual diet
Esposito et al. [31]	Italy	Parallel	MetS	EFS	180	44	28	135/86	96	MedDiet	Prudent diet
Fuentes et al. [32]	Spain	Crossover	Hypercholestrolemic	FMD, BVS	22	40	-	-	8	MedDiet + MUFA	NCEP-1 diet
Jaacks et al. [33]	USA	Parallel	Overweight	FMD	30	51	31.5	-	8	MedDiet	Habitual diet
Klonizakis et al. [34]	UK	Parallel	Healthy	CM	22	55	30.5	127/79	8	MedDiet + exercise	Non-MedDiet + exercise
Maiorino et al. [35]	Italy	Parallel	DM2	CIMT	215	52	29.6	140/87	121	MedDiet	Low-fat diet
Marin et al. [36]	Spain	Crossover	Healthy	CM	20	67	31.9	-	4	MedDiet	SFA diet
Murie-Fernandez et al. [37]	Spain	Parallel	CVD risk	CIMT	187	67	29.4	-	48	G1:MedDiet + EVOO	Low-fat diet
										G2: MedDiet + nuts	
Sala-Vila et al. [38]	Spain	Parallel	CVD risk	ICA-IMT	175	66	29.6	150/81	115	G1:MedDiet + EVOO	Low-fat diet
										G2: MedDiet + nuts	
Thomazella et al. [39]	Brazil	Parallel	ACS	FMD, BVS	42	55	26.4	136/84	12	MedDiet	Low-fat diet
Torres-Peña et al. [40]	Spain	Parallel	DM2	FMD	438	61	31.8	-	72	MedDiet + EVOO	Low-fat diet
Torres-Peña et al. [40]	Spain	Parallel	pDM2	FMD	289	58	30.3	-	72	MedDiet + EVOO	Low-fat diet
Torres-Peña et al. [40]	Spain	Parallel	Healthy	FMD	78	56	29.5	-	72	MedDiet + EVOO	Low-fat diet
Yubero-Serrano et al. [41]	Spain	Parallel	CHD	FMD	805	60	30.9	137/77	52	MedDiet	Low-fat diet

*ACS* acute coronary syndromes, *BVS* baseline vessel size, *CHD* coronary heart disease, *CIMT* carotid intima-media thickness, *CM* cutaneous microvascular function, *CVD risk* risk of cardiovascular disease, *DBP* diastolic blood pressure, *DM2* type 2 diabetes, *EFS* endothelial function score, *EVOO* extra virgin olive oil, *FBF* forearm blood flow, *FMD* flow-mediated dilation, *G1* group 1, *G2* group 2, *ICA-IMT* internal carotid intima-media thickness, *MedDiet* Mediterranean dietary pattern, *MetS* metabolic syndrome, *n* number of subjects, *NCEP-1* The National Cholesterol Education Program Diet, *pDM2* prediabetes, *SBP* systolic blood pressure

## Results

### Search results

**Fig. 1:**
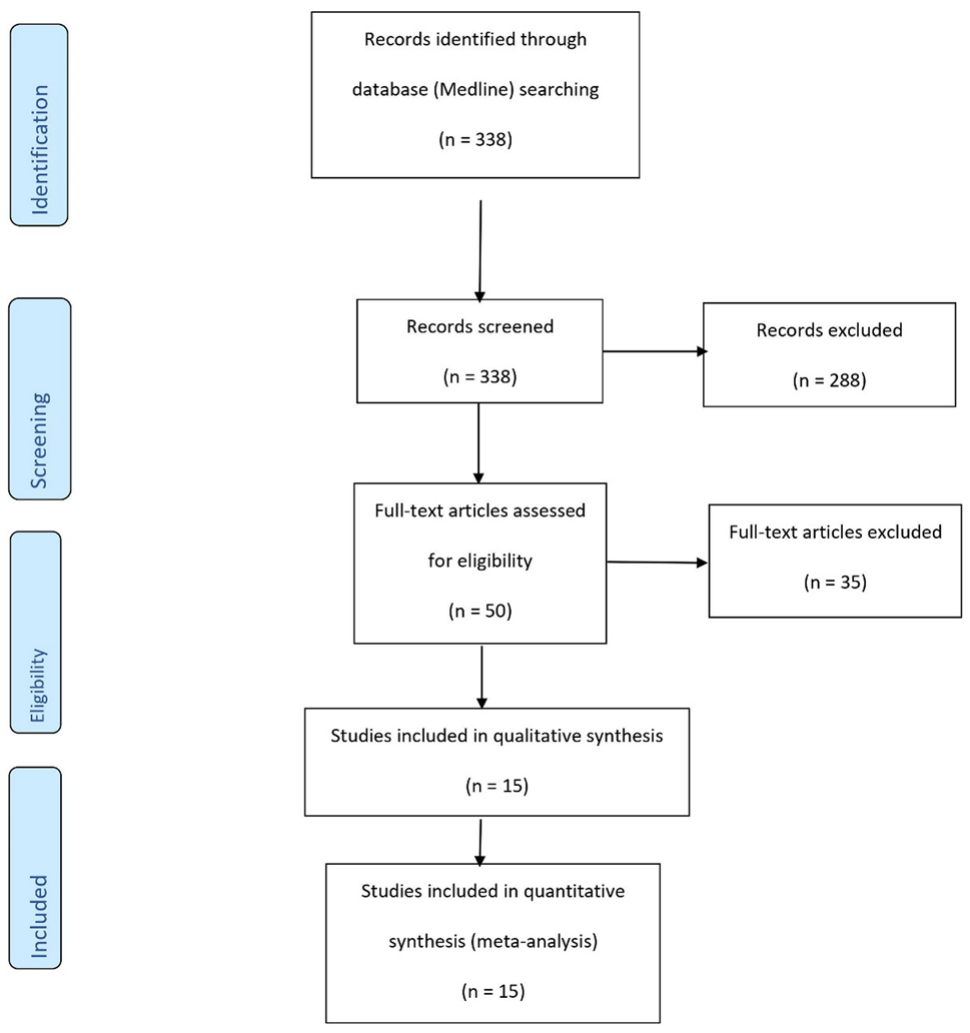
Flow diagram of the selection process of the randomized controlled trials included in the meta-analysis. EF, endothelial function; MD, Mediterranean diet

Our initial search on the Medline Database revealed 338 studies. The process is summarized is Fig. 1. After title and abstract screening, 50 studies were selected and the remaining were excluded. All 50 articles were assessed for full-text reading, out of which 15 articles were shortlisted for inclusion in our meta-analysis and systematic review, and 35 studies were ruled out because they did not meet our inclusion criteria. RCTs included in our study had results from independent studies that checked the effect of the MedDiet on endothelial function, producing a total of 21 sets of independent measures of endothelial function using different methods that were included in the meta-analyses.

### Study characteristics

Our meta-analysis included a total of 2735 participants from all 15 RCTs [20, 28–41] with a median of 182 (range 20–805) participants per study. The average participant age was 55 (range 20–75) years. Twelve out of 15 RCTs were parallel trials because some part of the population was tested with a control group and the remaining were crossover studies. The paired nature of crossover trials was taken into consideration to reduce the unit of analysis errors. The period of the intervention ranged from 1 to 30 months. Five studies examined the effect of the MedDiet on a healthy population [20, 28, 34, 36, 40], 3 in the diabetic population [30, 35, 40], 2 in people with an elevated risk of CVD [37, 38], 1 in patients with metabolic syndrome [20], 1 with pre-diabetics patients [40], 1 in patients with the acute coronary syndrome [39], 1 in hypercholesterolemic men [32], 1 in patients with coronary heart disease [40], 1 in obese patients [29], and 1 in overweight patients [33]. Various combinations of the MedDiet were employed such as a MedDiet only (*n* = 8), MedDiet plus supplementary nuts (*n* = 2), MedDiet plus supplementary extra virgin olive oil (*n* = 5), MedDiet plus MUFAs (*n* = 3), and MedDiet plus exercise (*n* = 1). Various control groups were also used, like a low-fat diet (*n* = 9), a Swedish diet (*n* = 1), the Atkins low-carbohydrate diet (*n* = 1), the participant’s normal diet (*n* = 2), the National Cholesterol Education Program Diet (*n* = 1), a non-MedDiet plus exercise (*n* = 1), saturated fatty acids (SFA) diet (*n* = 1), and a prudent diet (*n* = 1). In the included studies, there were several methods and parameters taken into account to investigate endothelial function, of which the most frequently used methods were FMD and carotid intima-media thickness. Some other parameters were also considered which included cutaneous microvascular function, FBF, baseline vessel size, and calculation of an endothelial function score.

### Meta-analysis

**Fig. 2:**
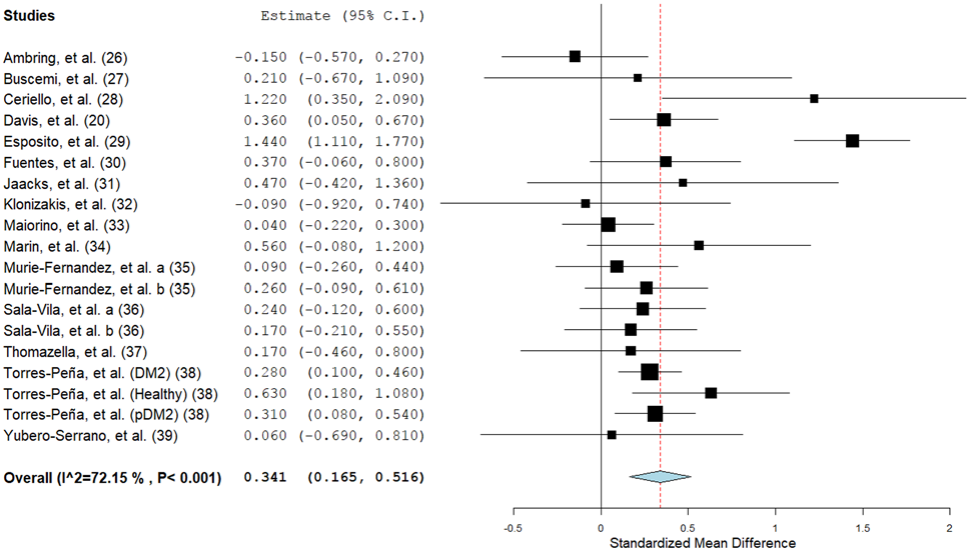
Forest plot showing overall effect of MedDiet on endothelial function. Data expressed as standardized mean difference (SMD)

**Fig. 3:**
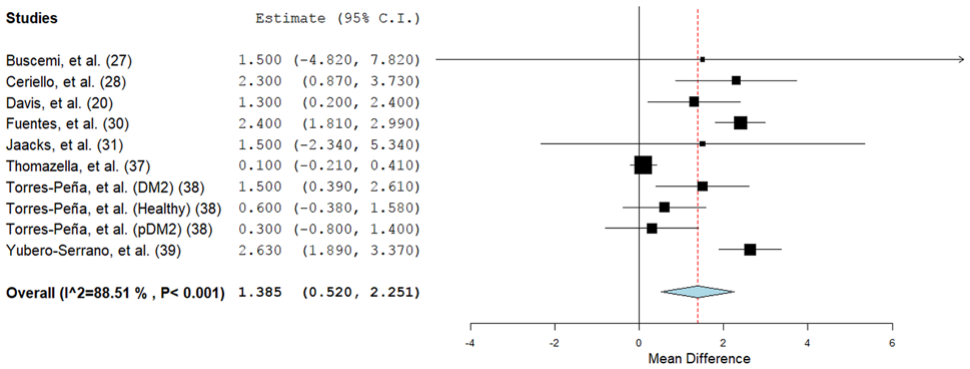
Forest plot showing the composite effect of MedDiet on flow-mediated dilation (expressed as percentage change)

**Fig. 4:**
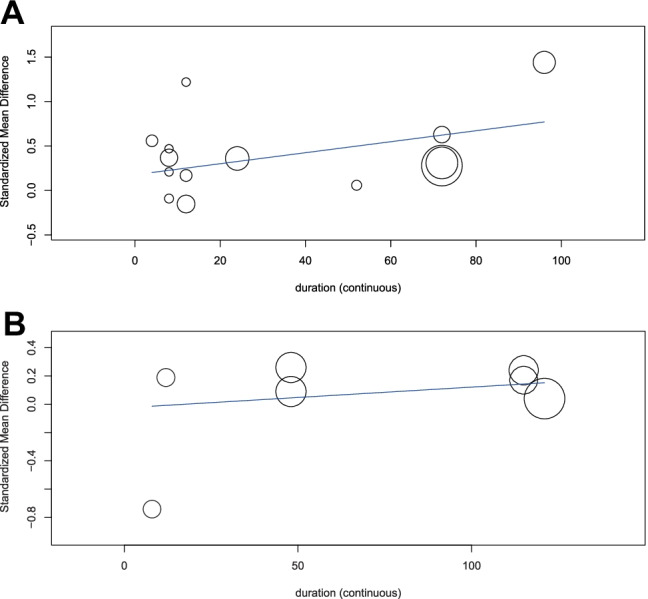
Meta-regression analysis of the association between study duration (weeks) and SMD of **a)** functional and **b)** structural parameters of endothelial function

With the inclusion of 21 sets of independent data, the meta-analysis showed that endothelial function improved, in general, with the intake of the MedDiet (SMD: 0.34; 95% CI: 0.16, 0.52; *P* = 0.0001; Fig. 2). There was a significant heterogeneity between the studies added (*Q* = 64.65; *I*^2^ = 72%; *P* < 0.00001). This heterogeneity of results was explained by the removal of 2 studies (Ambring et al., Esposito et al.) with wider effect estimates (*Q* = 14.00; *I*^2^ = 0%; *P* = 0.60), while still confirming a significant positive effect of the MedDiet on endothelial function (SMD: 0.27; 95% CI: 0.18, 0.36; *P* < 0.00001). Subgroup analyses indicated that the effect was stronger on functional parameters (SMD: 0.43; 95% CI: 0.19, 0.67; *P* = 0.0005; *I*^2^ = 76%), in comparison with structural parameters of endothelial function (SMD: 0.09; 95% CI: − 0.09, 0.27; *P* = 0.33; *I*^2^ = 34%). Overall, the MedDiet increased FMD by 1.39% (95% CI: 0.47, 2.19; *P* < 0.001; Fig. 3). Subgroup analysis also showed a significant improvement in endothelial function with the intake of MedDiet in both healthy participants (SMD: 0.29; 95% CI: 0.05, 0.53; *P* = 0.02; *I*^2^ = 27%) and in those with an increased likelihood of CVD (SMD: 0.29; 95% CI: 0.06, 0.52; *P* = 0.01; *I*^2^ = 81%). Moreover, there was no significant change (*p* = 0.22) in the effects of the MedDiet on endothelial function, due to the study design (crossover or parallel) or type of intervention (MedDiet alone or combined) (Table 2). Meta-regression analysis depicted no modification of the effect size by age (*p* = 0.618), BMI (*p* = 0.497), or study duration (in weeks) (*p* = 0.527) (eTable 1, *given in supplementary data*). Thus, it is confirmed that the impact of MedDiet on endothelial function was not influenced by age, BMI, or study duration. However, a significant association was demonstrated between study duration (weeks) and functional parameters (slope: 0.006; SE: 0.003; *P* = 0.053; Fig. 4a) but not with structural parameters (slope: 0.001; SE: 0.002; *P* = 0.401; Fig. 4b) of endothelial function.

**Table 2:** Sensitivity analysis to evaluate influence of health status, intervention type, measurement type and study design

**Category**	**No of EF measurements per subgroup**	**Effect Size**	**95% CI**	***p***	***P *between groups**	***I2***
1. Health Status					1	
Health	7	0.29	0.05–0.53	0.02		27%
Increased CVD risk	14	0.29	0.06–0.52	0.01		81%
2. Type of intervention					0.62	
MedDiet Only	10	0.35	0.03–0.73	0.07		83%
MedDiet + Other	11	0.25	0.08–0.41	0.003		50%
3. Type of measurement					0.003	
Structural	7	0.09	0.09–0.27	0.33		34%
Functional	14	0.43	0.19–0.67	< 0.001		76%
4. Study design					0.22	
Crossover	4	0.02	0.49–0.52	0.95		75%
Parallel	17	0.35	0.17–0.54	< 0.001		73%

### Study quality and publication bias

Overall, the trials were of moderate quality. A majority of studies did not properly report the presence of bias. Half of the studies indicated an attribution bias [20, 31, 33, 35, 38–40]. Only a limited number of studies reported a selection bias [33]. Details of the randomization procedure were mentioned in some studies [20, 29, 31, 34–36, 40] whereas details about the allocation concealment were indicated in 3 studies [31, 35, 36]. Records about any participants leaving or underreporting of the results were mentioned in 6 studies [28–30, 34, 36, 41]. In 3 studies [33, 36, 39], selective reporting of the outcome was well described. Summary and graph of study quality and risk assessment are given in Supplementary Fig. 1.

## Discussion

Overall, with the inclusion of 15 studies involving 2735 participants, this meta-analysis shows that a MedDiet can improve endothelial function as we observed favorable effects in both healthy individuals and those with an increased likelihood of CVD. Furthermore, subgroup analyses showed that the effect was stronger on functional parameters of endothelial function in comparison to structural parameters. A previous meta-analysis evaluating this association reported a 40% lower risk of CVD following MedDiet [42], whereas another study suggested a reduction in CVD risk following a MedDiet to be approximately 24% stronger compared to the respective control groups [43]. In preceding systematic reviews and meta-analyses, Shannon et al. [44] reported positive results with a MedDiet intervention. In particular, the meta-analysis highlighted that the MedDiet increased FMD by 1.66%, which reduced CVD risk by ~ 22%. This was calculated using the findings predicted by Inaba et al. [45] that a 13% decrease in the risk of cardiovascular events occurs per 1% increase in FMD [44]. Thus, with a 1.39% increase in FMD in our meta-analysis, the decrease in risk of CVD is approximately 18%.

The MedDiet can reduce the risk of CVD by improving endothelial function through several different mechanisms such as by (a) protecting against oxidative stress, inflammation, and platelet aggregation (b) modifying cancer-related hormones and growth factors (c) by having a lipid-lowering effect, and (d) by modulating gut microbiota-mediated production of metabolites influencing metabolic health [46]. Additionally, it can significantly decrease blood lipid levels of low-density lipoprotein (LDL), apolipoprotein B, and cholesterol which ultimately improves endothelial function. Oxidized LDL promotes endothelial dysfunction as it plays an important role in creating an environment for inflammation and lipid accumulation within vessels, which can lead to the development of atherosclerosis [47]. The MedDiet improves vasomotor function and decreases LDL cholesterol levels by decreasing P-selectin in plasma. In addition, MUFA, a major component of MedDiet, also improves endothelial function in hypercholesterolemic men, thus reducing the risk of atherosclerotic plaque formation and decreasing cholesterol levels [32]. Studies have also shown that MedDiet increases high-density lipoprotein-cholesterol (HDL-C) plasma levels while decreasing LDL oxidation so it is used for both primary and secondary prevention of CVD [48]. HDL has many cardioprotective benefits; its plasma levels increase due to the Mediterranean diet rich in olive oil [49].

The MedDiet is a complex diet enriched by foods such as nuts, legumes, and fish. All of which are key sources of l-arginine, an amino acid that can be utilized to enhance nitric oxide (NO) bioavailability. NO accounts for the relaxation of vascular tone [4, 50] and inhibition of platelet aggregation [51] as well as platelet adhesion [52]. The importance of l-arginine in amounts 4–24 g per day can be seen through cardiovascular benefits such as reduced BP wherein systolic and diastolic BP can decrease by approximately 5- and 3-mm Hg, respectively. Additionally, nitrate is also an important precursor for NO production and can be found in vegetables, which are included in the MedDiet; nitrate also presents with similar benefits [53].

Another known effect of the MedDiet is on inflammatory markers. Prior studies have reported that some components of the MedDiet such as nuts may downregulate inflammatory markers related to atherosclerosis, such as serum C-reactive protein (CRP), interleukin 6 (IL-6), cell adhesion molecule-1 (CAM-1), and intercellular adhesion molecule-1 (ICAM-1) [54]. For example, CRP reduces endothelial nitric oxide synthase (eNOS) activity which subsequently reduces vasodilator effects of NO and increases vasoconstrictor effects of Endothelin-1 (ET-1) all of which increases the chances of atherosclerosis and clot formation [42]. This downregulation of inflammatory markers leads to improvements in endothelial function.

The overall quality of the studies involved in our meta-analysis was moderate. In most RCTs, the participants were aware of the intervention while the information was kept confidential in a few studies. This can be viewed as a notable limitation, keeping in mind that expectation bias can play its part, where the anticipation of beneficial effects can produce the most advantageous outcome. At the same time, it has been observed that blinding patients in a clinical trial, in which the intervention is a diet, is a difficult task to achieve. In some of the studies, analysis of endothelial function was done more than once during the trial period, so for studies with a longer period of intervention, this may have impacted the results. To eliminate doubts of having biases in our study related to intermediate measurements of endothelial function, we opted for a systematic approach by including only the last measurement in our study.

We revised previous meta-analysis and offer the most recent data. Our meta-analysis results are similar with earlier meta-analysis findings, bolstering the notion that MedDiet is helpful in decreasing CVD and FMD risk values. Furthermore, our findings open the door to additional research that should be conducted to evaluate the efficacy of MedDiet in structural aspects of endothelial function using more robust trials. Our findings could aid in the development of MedDiet diet plans and will help improve the guidelines for patients with CVD. A limitation of our research was that the mean age of participants was 50 years old, which may have minimally decreased the impact fullness of our results, although meta-regression negates the linkage of effects of the MedDiet with age. Furthermore, because there is no general definition of what defines a MedDiet, the nature of the dietary interventions varied across research, and it is probable that certain MedDiet variations may be more beneficial than others in terms of increasing endothelial function, as evidenced by the substantial degree of heterogeneity in our analyses. Similarly, the control group used had variation that there was no a consistent standard against which the MedDiet was measured, which could lead to the high heterogeneity in our analysis. We only considered those studies in our meta-analysis in which the author mentioned intervention as the MedDiet; therefore, we may have missed some studies in which the MedDiet was directed at patients without using the actual name of the diet, and therefore this can be considered a methodological limitation. More studies are required to evaluate the effect of MedDiet on endothelial function, with larger cohorts and longer durations. More robust trials are required to assess the disparities in effects on MedDiet on endothelial function based on gender, age, and previous history of comorbidities so that more rigorous analysis can be performed to better understand the association between MedDiet and endothelial function.

## Conclusion

The current meta-analysis depicts that the MedDiet has positive effects on cardiovascular health by improving the functional and structural parameters of endothelial function. We did not observe any variation due to age, BMI, study duration, or study design in the effects of MedDiet on endothelial function. Moreover, the quality of evidence in our research was modest but future clinical trials with a good sample size and age diversity are required to strengthen the proof that the MedDiet has beneficial cardiovascular health effects.

## Supplementary information

Below is the link to the electronic supplementary material.

Supplementary file1 (DOCX 150 KB)
