# Supplementary material for: Mediterranean Diet and its Effect on Endothelial Function: A Meta-analysis and Systematic Review
Source: Ir J Med Sci. 2022 Feb 22;192(1):105–13. doi: 10.1007/s11845-022-02944-9 (PMC9892125; doi:10.1007/s11845-022-02944-9)
Supplement: Supplementary file 1 — Supplementary file1 (DOCX 150 KB) [file 11845_2022_2944_MOESM1_ESM.docx]

**Supplementary Methods 1**

| **Search String:**  (("mediterranean"[All Fields] OR "mediterraneans"[All Fields]) AND "diet*"[All Fields] AND (("Flow-mediated"[All Fields] AND ("dilatable"[All Fields] OR "dilatated"[All Fields] OR "dilatating"[All Fields] OR "dilatation"[MeSH Terms] OR "dilatation"[All Fields] OR "dilatations"[All Fields] OR "dilate"[All Fields] OR "dilation"[All Fields] OR "dilations"[All Fields] OR "dilatative"[All Fields] OR "dilatator"[All Fields] OR "dilatators"[All Fields] OR "dilated"[All Fields] OR "dilates"[All Fields] OR "dilating"[All Fields] OR "dilator"[All Fields] OR "dilators"[All Fields])) OR ("Flow-mediated"[All Fields] AND ("dilatable"[All Fields] OR "dilatated"[All Fields] OR "dilatating"[All Fields] OR "dilatation"[MeSH Terms] OR "dilatation"[All Fields] OR "dilatations"[All Fields] OR "dilate"[All Fields] OR "dilation"[All Fields] OR "dilations"[All Fields] OR "dilatative"[All Fields] OR "dilatator"[All Fields] OR "dilatators"[All Fields] OR "dilated"[All Fields] OR "dilates"[All Fields] OR "dilating"[All Fields] OR "dilator"[All Fields] OR "dilators"[All Fields])) OR "FMD"[All Fields] OR (("veins"[MeSH Terms] OR "veins"[All Fields] OR "venous"[All Fields]) AND ("dental occlusion"[MeSH Terms] OR ("dental"[All Fields] AND "occlusion"[All Fields]) OR "dental occlusion"[All Fields] OR "occlusion"[All Fields] OR "occlused"[All Fields] OR "occlusions"[All Fields] OR "occlusive"[All Fields] OR "occlusives"[All Fields]) AND ("plethysmography"[MeSH Terms] OR "plethysmography"[All Fields] OR "plethysmographies"[All Fields])) OR (("peripheral"[All Fields] OR "peripherally"[All Fields] OR "peripherals"[All Fields] OR "periphereal"[All Fields] OR "peripheric"[All Fields] OR "peripherically"[All Fields]) AND ("arterialization"[All Fields] OR "arterializations"[All Fields] OR "arterialize"[All Fields] OR "arterialized"[All Fields] OR "arterializing"[All Fields] OR "arterially"[All Fields] OR "arterials"[All Fields] OR "arterie"[All Fields] OR "arteries"[MeSH Terms] OR "arteries"[All Fields] OR "arterial"[All Fields] OR "arteris"[All Fields] OR "artery"[All Fields] OR "arterious"[All Fields] OR "artery s"[All Fields] OR "arterys"[All Fields]) AND ("manometry"[MeSH Terms] OR "manometry"[All Fields] OR "tonometry"[All Fields] OR "tonometries"[All Fields])) OR ("nitric oxide"[MeSH Terms] OR ("nitric"[All Fields] AND "oxide"[All Fields]) OR "nitric oxide"[All Fields]) OR (("endothelialization"[All Fields] OR "endothelialize"[All Fields] OR "endothelialized"[All Fields] OR "endothelializing"[All Fields] OR "endothelials"[All Fields] OR "endothelium"[MeSH Terms] OR "endothelium"[All Fields] OR "endothelial"[All Fields]) AND ("functional"[All Fields] OR "functional s"[All Fields] OR "functionalities"[All Fields] OR "functionality"[All Fields] OR "functionalization"[All Fields] OR "functionalizations"[All Fields] OR "functionalize"[All Fields] OR "functionalized"[All Fields] OR "functionalizes"[All Fields] OR "functionalizing"[All Fields] OR "functionally"[All Fields] OR "functionals"[All Fields] OR "functioned"[All Fields] OR "functioning"[All Fields] OR "functionings"[All Fields] OR "functions"[All Fields] OR "physiology"[MeSH Subheading] OR "physiology"[All Fields] OR "function"[All Fields] OR "physiology"[MeSH Terms])) OR (("endothelialization"[All Fields] OR "endothelialize"[All Fields] OR "endothelialized"[All Fields] OR "endothelializing"[All Fields] OR "endothelials"[All Fields] OR "endothelium"[MeSH Terms] OR "endothelium"[All Fields] OR "endothelial"[All Fields]) AND ("dysfunctional"[All Fields] OR "dysfunctionals"[All Fields] OR "dysfunctioning"[All Fields] OR "dysfunctions"[All Fields] OR "physiopathology"[MeSH Subheading] OR "physiopathology"[All Fields] OR "dysfunction"[All Fields])) OR ("carotid intima media thickness"[MeSH Terms] OR ("carotid"[All Fields] AND "intima media"[All Fields] AND "thickness"[All Fields]) OR "carotid intima media thickness"[All Fields] OR ("carotid"[All Fields] AND "intima"[All Fields] AND "media"[All Fields] AND "thickness"[All Fields]) OR "carotid intima media thickness"[All Fields]) OR ("pulse wave analysis"[MeSH Terms] OR ("pulse"[All Fields] AND "wave"[All Fields] AND "analysis"[All Fields]) OR "pulse wave analysis"[All Fields] OR ("pulse"[All Fields] AND "wave"[All Fields] AND "velocity"[All Fields]) OR "pulse wave velocity"[All Fields]) OR (("augment"[All Fields] OR "augmentation"[All Fields] OR "augmentations"[All Fields] OR "augmented"[All Fields] OR "augmenting"[All Fields] OR "augments"[All Fields]) AND ("abstracting and indexing"[MeSH Terms] OR ("abstracting"[All Fields] AND "indexing"[All Fields]) OR "abstracting and indexing"[All Fields] OR "index"[All Fields] OR "indexed"[All Fields] OR "indexes"[All Fields] OR "indexing"[All Fields] OR "indexation"[All Fields] OR "indexations"[All Fields] OR "indexe"[All Fields] OR "indexer"[All Fields] OR "indexers"[All Fields] OR "indexs"[All Fields])))) |
| --- |

**Supplementary Figure 1: Risk of Bias Assessment**

**
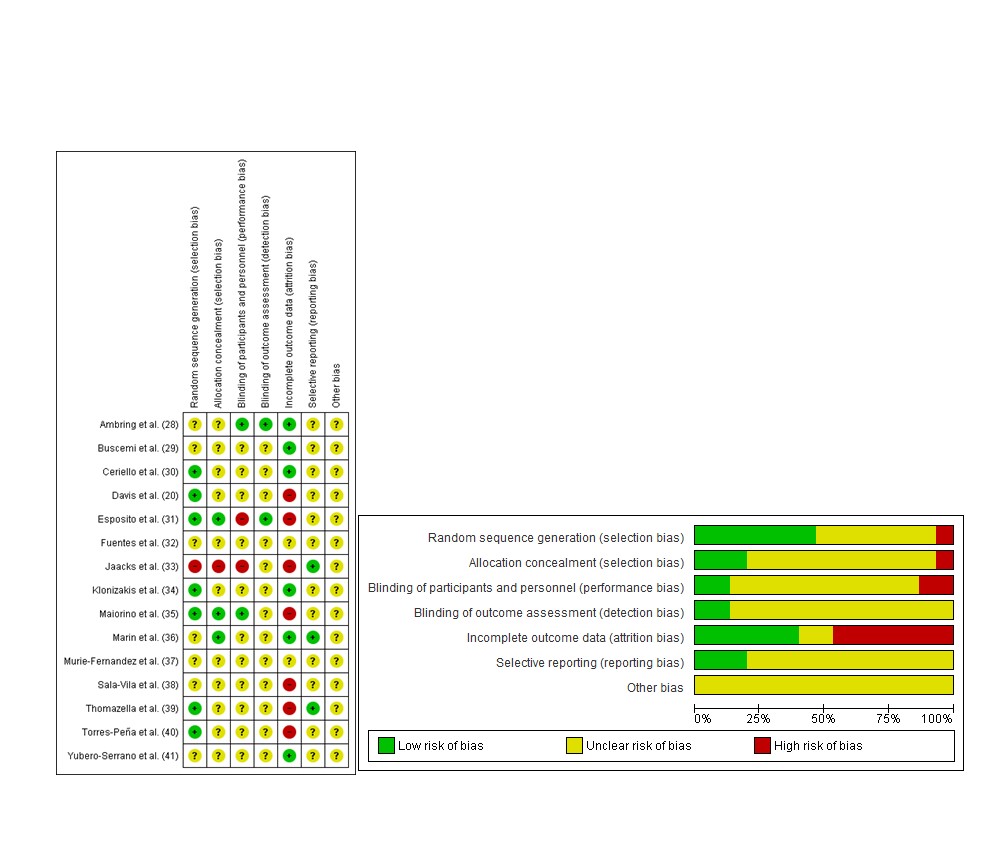
**

**eTable 1:** Meta-regression analysis to evaluate effect of age, BMI and study duration

|  | Slope | SE | p-value |
| --- | --- | --- | --- |
| Age, y | -0.004 | 0.009 | 0.618 |
| Study duration, week | 0.001 | 0.002 | 0.527 |
| BMI, kg/m | -0.032 | 0.047 | 0.497 |

**Supplemental Table 1: Raw data used in meta-analysis**

| **Reference** | **Subgroup within study** | **Comparison** | **Outcome** | **Data format** | **Age**  **(yrs)** | **BMI**  **(kg/m^2^)** | **Duration**  **(weeks)** | **Design** | **Type**  **Of**  **MD** | **Difference in means** | **N** | **Paired groups**  **p-value** | **Tails** |
| --- | --- | --- | --- | --- | --- | --- | --- | --- | --- | --- | --- | --- | --- |
| **Ambring et al. (28)** | **Healthy** | **Functional** | **FBF** | **Paired groups (difference, p)** | **43** | **26** | **12** | **Cross-over** | **MD** | **0.41** | **22** | **0.5** | **2** |
| **Fuentes et al. (32)** | **Increased CVD risk** | **Functional** | **FMD** | **Paired groups (difference, p)** | **40** | **28** | **8** | **Cross-over** | **MDPlus** | **2.4** | **22** | **0.05** | **2** |
| **Fuentes et al. (32)** | **Increased CVD risk** | **Structural** | **BVS** | **Paired groups (difference, p)** | **40** | **28** | **8** | **Cross-over** | **MDPlus** | **0.02** | **22** | **0.18** | **2** |
| **Yubero-Serrano et al. (41)** | **Increased CVD risk** | **Functional** | **FMD** | **Paired groups (difference, p)** | **60.4** | **30.9** | **52** | **Parallel** | **MD** | **2.63** | **805** | **0.011** | **2** |

**Supplemental Table 2: Raw data used in meta-analysis**

| **Reference** | **Subgroup within study** | **Comparison** | **Outcome** | **Data Format** | **Age**  **(yrs)** | **BMI**  **(kg/m^2^)** | **Duration**  **(weeks)** | **Design** | **Type**  **Of**  **MD** | **Difference in means** | **Lower Limit** | **Upper Limit** | **MD**  **n** | **Control**  **n** | **Confidence Level** |
| --- | --- | --- | --- | --- | --- | --- | --- | --- | --- | --- | --- | --- | --- | --- | --- |
| **Davis et al. (20)** | **Healthy** | **Functional** | **FMD** | **Raw Difference (independent groups, CI)** | **71** | **26.9** | **24** | **Parallel** | **MD** | **1.3** | **0.2** | **2.4** | **85** | **81** | **0.95** |

**Supplemental Table 3**

| **Reference** | **Subgroup within**  **study** | **Comparison** | **Outcome** | **Data format** | **Age** | **BMI** | **Duration** | **Design** | **Typeof**  **MD** | **MD**  **Pre Mean** | **MD**  **Pre SD** | **MD**  **Post Mean** | **MD**  **Post SD** | **MD**  **N** | **Control Pre Mean** | **Control Pre SD** | **Control Post Mean** | **Control**  **Post SD** | **Control**  **N** | **Pre Post**  **correlation** |
| --- | --- | --- | --- | --- | --- | --- | --- | --- | --- | --- | --- | --- | --- | --- | --- | --- | --- | --- | --- | --- |
| **Buscemi, et**  **al. (29)** | **Healthy** | **Functional** | **FMD** | **Means,**  **SD in each** | **38** | **34.2** | **8** | **Parallel** | **MD** | **10.3** | **7** | **10.6** | **6** | **10** | **12.2** | **9** | **11** | **4** | **10** | **0.5** |
| **Ceriello, et**  **al. (30)** | **Increased**  **CVD Risk** | **Functional** | **FMD** | **Means,**  **SD in each** | **71** | **29.5** | **12** | **Parallel** | **MDPlus** | **5.6** | **2** | **7.9** | **1.4** | **1 2** | **5.5** | **1** | **5.6** | **2.1** | **12** | **0.5** |
| **Esposito, et**  **al. (31)** | **Increased**  **CVD Risk** | **Functional** | **EFS** | **Means,**  **SD in each group** | **44** | **25** | **96** | **Parallel** | **MD** | **6** | **1.2** | **7.9** | **1.3** | **90** | **5.9** | **1.1** | **6.1** | **1.1** | **90** | **0.5** |
| **Jaacks**  **et al. (33)** | **Healthy** | **Functional** | **FMD** | **Means,**  **SD in each**  **group** | **51** | **31.5** | **8** | **Parallel** | **MD** | **5.6** | **3.3** | **6.7** | **3.6** | **11** | **6.1** | **5.3** | **5.2** | **4.9** | **9** | **0.5** |
| **Klonizakis**  **et al. (34)** | **Healthy** | **Functional** | **CM** | **Means,**  **SD in each group** | **55** | **30.4** | **8** | **Parallel** | **MDPlu**  **s** | **0.74** | **0.2**  **6** | **2.04** | **1.1** | **11** | **0.86** | **0.44** | **2.27** | **1 .67** | **11** | **0.5** |
| **Thomazella**  **et al. (39)** | **Increased**  **CVD Risk** | **Functional** | **FMD** | **Means,**  **SD in each** | **55** | **26.4** | **12** | **Parallel** | **MD** | **4.4** | **3.6** | **4.9** | **4.3** | **21** | **4.4** | **5.5** | **49** | **3.7** | **19** | **0.5** |
| **Thomazella**  **et al. (39)** | **Increased**  **CVD Risk** | **Structural** | **BVS** | **Means,**  **SD in each group** | **55** | **26.4** | **12** | **Parallel** | **MD** | **4.38** | **0.3**  **9** | **4.44** | **0.5**  **2** | **21** | **4.44** | **0.51** | **4.34** | **0.49** | **19** | **0.5** |
| **Torres-Pena**  **et al. (DM2) (40)** | **Increased**  **CVD Risk** | **Functional** | **FMD** | **Means,**  **SD in each group** | **61** | **32** | **72** | **Parallel** | **MDPlus** | **3.8** | **5.9** | **5.2** | **5.9** | **220** | **3.98** | **5.9** | **3.7** | **5.9** | **218** | **0.5** |
| **Torres-Pena**  **et al. (pDM2) (40)** | **Increased**  **CVD Risk** | **Functional** | **FMD** | **Means,**  **SD in each group** | **58** | **30.3** | **72** | **Parallel** | **MDPlus** | **3.8** | **4.9**  **6** | **4.9** | **4.9**  **6** | **154** | **5.1** | **5.8** | **4.6** | **46** | **135** | **0.5** |
| **Torres-Pena**  **et at. (Healthy) (40)** | **Healthy** | **Functional** | **PMD** | **Means,**  **SD in each group** | **56** | **29.5** | **72** | **Parallel** | **MDPlu**  **s** | **4.8** | **3.2** | **4.2** | **2.6**  **5** | **44** | **6.1** | **3.5** | **3.6** | **1.75** | **34** | **0.5** |

**Supplemental Table 4: Raw data used in meta-analysis**

| **Reference** | **Subgroup within**  **study** | **Comparison** | **Outcome** | **Data format** | **Age** | **BMI** | **Duration** | **Design** | **Type of**  **MD** | **MD**  **Mean Difference** | **MD**  **Different e SD** | **MD**  **n** | **Control**  **Mean Difference** | **Control**  **Difference SD** | **Control**  **n** | **Pre Post**  **correlation** |
| --- | --- | --- | --- | --- | --- | --- | --- | --- | --- | --- | --- | --- | --- | --- | --- | --- |
| **Maiorino et al. (35)** | **Increase**  **d CVD**  **Risk** | **Structural** | **CIMT** | **Mean**  **change, SD**  **difference in each group** | **52** | **29.6** | **121** | **Parallel** | **MD** | **0.026** | **0.727** | **108** | **0.001** | **0.62** | **107** | **0.5** |
| **Murie-Fernandez et al. a**  **(37)** | **Increase**  **d CVD**  **Risk** | **Structural** | **CIMT** | **Mean**  **change, SD**  **difference in each** | **67** | **29.4** | **48** | **Parallel** | **MDPlus** | **-0.016** | **0.11** | **66** | **-0.007** | **0.09** | **62** | **0.5** |
| **Murie-Fernandez et al. b**  **(37)** | **Increase**  **d CVD**  **Risk** | **Structural** | **CIMT** | **Mean**  **change, SD**  **difference in each** | **67** | **29.4** | **48** | **Parallel** | **MDPlus** | **-0.03** | **0.09** | **59** | **-0.007** | **0.09** | **62** | **0.5** |
| **Sala-Vila et al. a (38)** | **Increase**  **d CVD**  **Risk** | **Structural** | **ICA-**  **IMT** | **Mean**  **change, SD**  **difference in each group** | **6fi** | **29.6** | **115** | **Parallel** | **MDPlus** | **-0.028** | **0.1** | **57** | **-0.004** | **0.1** | **61** | **0.5** |
| **Sala-Vila et at. B (38)** | **Increase**  **d CVD**  **Risk** | **Structural** | **IDA-**  **IMT** | **Mean**  **change, SD**  **difference in each** | **66** | **29.6** | **115** | **Parallel** | **MDPlus** | **-0.021** | **0.1** | **46** | **-0.004** | **0.1** | **61** | **0.5** |

**Supplemental Table 5: Raw data used in meta-analysis**

| **Reference** | **Subgroup**  **within**  **study** | **Comparison** | **Outcome** | **Data**  **format** | **Age** | **BMI** | **Duration** | **Design** | **Type of**  **MD** | **MD**  **Mean** | **MD**  **SD** | **MD**  **n** | **Control**  **Mean** | **Control**  **SD** | **Control**  **n** |
| --- | --- | --- | --- | --- | --- | --- | --- | --- | --- | --- | --- | --- | --- | --- | --- |
| **Marin et al. (36)** | **Healthy** | **Functional** | **CM** | **Independent groups**  **(means, SD's)** | **70** | **25** | **4** | **Cross-Over** | **MD** | **88** | **61.3** | **20** | **59.6** | **36.2** | **20** |
